# Altered iron metabolism in cystic fibrosis macrophages: the impact of CFTR modulators and implications for *Pseudomonas aeruginosa* survival

**DOI:** 10.1038/s41598-020-67729-5

**Published:** 2020-07-02

**Authors:** H. F. Hazlett, T. H. Hampton, D. S. Aridgides, D. A. Armstrong, J. A. Dessaint, D. L. Mellinger, A. B. Nymon, A. Ashare

**Affiliations:** 1 Department of Medicine, Dartmouth-Hitchcock Medical Center, Geisel School of Medicine at Dartmouth, 1 Medical Center Dr, Lebanon, NH 03756 USA; 2 Microbiology and Immunology, Dartmouth College, Hanover, NH USA; 3 Department of Microbiology and Immunology, Geisel School of Medicine at Dartmouth, 1 Medical Center Dr, Lebanon, NH 03756 USA

**Keywords:** Biofilms, Monocytes and macrophages, Cystic fibrosis

## Abstract

Cystic fibrosis (CF) is a genetic disease caused by mutations in the CF transmembrane conductance regulator (*CFTR*) gene, resulting in chronic bacterial lung infections and tissue damage. CF macrophages exhibit reduced bacterial killing and increased inflammatory signaling. Iron is elevated in the CF lung and is a critical nutrient for bacteria, including the common CF pathogen *Pseudomonas aeruginosa* (*Pa*). While macrophages are a key regulatory component of extracellular iron, iron metabolism has yet to be characterized in human CF macrophages. Secreted and total protein levels were analyzed in non-CF and F508del/F508del CF monocyte derived macrophages (MDMs) with and without clinically approved CFTR modulators ivacaftor/lumacaftor. CF macrophage transferrin receptor 1 (TfR1) was reduced with ivacaftor/lumacaftor treatment. When activated with LPS, CF macrophage expressed reduced ferroportin (Fpn). After the addition of exogenous iron, total iron was elevated in conditioned media from CF MDMs and reduced in conditioned media from ivacaftor/lumacaftor treated CF MDMs. *Pa* biofilm formation and viability were elevated in conditioned media from CF MDMs and biofilm formation was reduced in the presence of conditioned media from ivacaftor/lumacaftor treated CF MDMs. Defects in iron metabolism observed in this study may inform host–pathogen interactions between CF macrophages and *Pa*.

## Introduction

Cystic fibrosis (CF) is a genetic disease that afflicts approximately 31,000 people in the United States, with a median predicted survival of 47.4 years for those born in 2018 (CF Foundation Registry Annual Report, 2018). CF is caused by mutations in the CF transmembrane conductance regulator (*CFTR*) gene, a chloride and bicarbonate channel that regulates epithelial anion transport^1^. While over 1,800 CFTR mutations have been identified, the F508del mutation is the most prevalent: 44.2% of the population is F508del homozygous (CF Foundation Registry Annual Report, 2018). Defective chloride and bicarbonate secretion, coupled with continued absorption of sodium by the epithelial sodium channel (ENaC), facilitates an accumulation of dehydrated mucous in the airway. Bicarbonate aids in the maintenance of airway pH and facilitates antimicrobial peptide function^2^, and disruption of bicarbonate contributes to airway acidification and reduced bacterial clearance in CFTR knockout pigs^3^. Additionally, increased mucin content is associated with neutrophil recruitment and inflammatory markers in the bronchoalveolar lavage of young children with CF^4^ and CFTR knockout ferrets^5^. These changes in airway pH, bacterial clearance, and inflammatory milieu facilitate chronic lung disease and bacterial infection over the lifetime of the CF patient^6^.

One of the most common and deleterious opportunistic pathogens that colonize the adult CF lung is *Pseudomonas aeruginosa* (*Pa*)^7–9^. As of 2018, 45.3% of CF subjects in the United States were reported to have *Pa* cultured from their sputum and 16.9% of CF subjects with a *Pa* infection were positive for multidrug-resistant *Pa* (CF Foundation Registry Annual Report, 2018). As a mechanism for survival in the airways, *Pa* form surface-attached communities, known as biofilms, in CF lung tissue and sputum^10^. Over the course of biofilm formation *Pa* become sessile and develop protective complexes rich in polysaccharides and proteins that allow the bacteria to evade phagocytosis and persist in the presence of antibiotics^10^.While most bacteria require iron to survive, *Pa* is an organism with a high requirement for iron whose biofilm formation can be influenced by the presence or absence of iron^11–14^. In clinically stable CF subjects, the amount of *Pa* recovered from the sputum strongly correlates with sputum iron levels^15^. CF airways have elevated levels of iron due in large part to tissue damage^15,16^. In addition to the correlations found between *Pa* recovery and airway iron levels, in vitro models of CF airway epithelium have demonstrated that iron availability is a critical factor in *Pa* biofilm formation^13,17^.

Mounting evidence suggests that perturbation of the CF immune response contributes to a vicious cycle of infection, inflammation, and neutrophil recruitment that culminates in airway tissue damage^18,19^. While there are a multitude of factors that contribute to CF disease progression, lung macrophages play a critical role in maintaining homeostasis in the airway. In addition to being integral phagocytic first responders to airway pathogens such as *Pa*, lung macrophages regulate and coordinate the inflammatory milieu and neutrophil recruitment^20^. Both extrinsic (pulmonary microenvironment) and intrinsic (CFTR expression) factors inform the CF macrophage immunophenotype^21–23^. Examples of CF macrophage dysfunction in primary human samples as well as in various CF animal models have been increasingly described throughout the literature. For example, macrophages isolated from CF patients express less CD206 and fail to polarize towards an “alternative” activation state in the presence of interleukin (IL)-13, suggesting defective signaling pathways related to the resolution of inflammatory signaling^24^. To compound this, CF macrophages exhibit a hyper-inflammatory response to a range of antigens and pathogens^23,25,26^. In addition to an altered cytokine profile and activation state, CF macrophages exhibit reduced phagocytic and bactericidal capacity^27^. Finally, CFTR dysfunction perturbs mitochondrial activity, anti-bacterial metabolite production, and reactive oxygen species (ROS) generation^28,29^, demonstrating broad implications for CF macrophage dysfunction.

There are several host cells, including hepatocytes, epithelial cells, erythrocytes, and macrophages, that play an important role to balance the uptake, secretion, and storage of iron^30,31^. In macrophages and other iron-regulatory cell types, there are specific programs of iron-regulation that are implemented during infection and inflammation. Macrophages in particular play an integral role in sensing and regulating iron, and have been increasingly recognized as critical regulators of iron availability in the local microenvironment during infection, inflammation, and wound healing^30–32^. In response to extracellular pathogens and inflammatory cytokines, macrophages and neutrophils secrete lactoferrin and lipocalin 2, proteins that respectively bind free iron and iron-loaded bacterial siderophores to directly scavenge iron from pathogens^33^. Extracellular iron is imported by transferrin receptor 1, which becomes endocytosed upon binding to iron-bound transferrin^36,37^. The inflammatory cytokine IL-6 activates production of the antimicrobial hormone hepcidin, which binds to and induces ubiquitination of the iron exporter ferroportin^38,39^, preventing iron export back into the microenvironment. When not immediately used or exported by ferroportin, intracellular iron is stored in ferritin complexes^40,41^. The appropriate storage of iron is important because free iron (II) reacts with hydrogen peroxide to produce hydroxyl free radicals in a process known as the Fenton reaction^42^. Anti-inflammatory anti-oxidants like heme oxygenase 1 (HO-1) are upregulated to reduce intracellular oxidative stress and negatively regulate macrophage NfκB signaling via toll-like receptor (TLR)4^43–47^.

The host iron metabolism can influence the course of infection and inflammation in multiple ways. In macrophages, iron is a critical nutrient for intracellular processes, including mitochondrial respiration^48^ and TLR4 signaling^39,49^. Dysregulation of iron-regulatory proteins like HO-1 contribute to macrophage hyperinflammation, intracellular oxidative stress, and apoptosis^18,45,46^. Additionally, elevated iron in the microenvironment can facilitate biofilm formation and growth of *Pa*^13,17^. Several publications have shown that perturbation of iron metabolism may impact host–pathogen interactions in the context of CF and *Pa* specifically, as CFTR mutation in bronchial epithelial cells (CFBEs) has been found to contribute to *Pa* survival via increased biofilm formation^13,17^.

While CFTR function in bronchial epithelial cells is well characterized, the precise role played by CFTR in human macrophages is complex and remains the subject of ongoing investigation^28^. Regardless, the importance of functional CFTR in macrophages has been demonstrated in studies analyzing CFTR modulator-corrected CF macrophage dysfunction^27,50^. CFTR modulators increase the phagocytic capacity in CF macrophages, alter CF macrophage cytokine production, and alter CF monocyte activation^27,50,51^. The impact of CFTR mutation, and its resulting host environment, on human macrophage iron metabolism remains unknown. We hypothesized that (1) iron regulatory pathways are dysfunctional in F508del/F508del CF MDMs in a manner relevant to host–pathogen interactions, and (2) ex vivo ivacaftor/lumacaftor treatment corrects iron regulatory pathway dysfunction.

## Methods

### Human monocyte isolation and differentiation

Non-CF and CF subject between 18 and 45 years of age were enrolled in the study. Non-CF subjects were recruited by flyers posted on the Dartmouth campus. Exclusion criteria for non-CF volunteers: upper respiratory infection (URI) symptoms within 14 days of blood draw, antibiotics within 28 days of blood draw, current smoker, any inhaled medication, chronic steroid use, use of any immunomodulatory drug, pregnancy, any history of respiratory disease, autoimmune condition, cardiac disease, neurologic disease, or other serious medical condition. CF subjects were excluded if they had exacerbation symptoms currently or had been treated with oral or IV antibiotics over the past 28 days or were on prednisone. CF subjects who did not have the F508del/F508del mutation were excluded from the study. In CF subjects, the use of maintenance inhaled antibiotics, pancreatic enzymes, vitamins, or other necessary medications were not exclusion criteria. Following informed consent, subjects underwent phlebotomy. This study was approved by the Institutional Review Board at Dartmouth-Hitchcock, protocol number 22781, and all methods were carried out in accordance with relevant guidelines and regulations. As described previously^27^, monocytes were isolated from whole blood within one hour of phlebotomy via density gradient centrifugation and negatively enriched by magnetic column (Miltenyi Biotec, cat#130-096-537). Monocytes were seeded in RPMI 1640 Glutamax (Gibco, cat#61870-036) with 50 µg/ml gentamicin (Caisson Laboratories, cat#ABL03-10ML) at 5 × 10^5^ cells/well in 12-well culture plates and incubated for 2 h to adhere. Monocytes were differentiated into monocyte derived macrophages (MDMs) for 7 days in RPMI 1640 Glutamax with 50 µg/ml gentamicin with 10% heat-inactivated FBS (HI-FBS; Genesee Scientific, cat#25-514H) with 100 ng/ml M-CSF (Miltenyi, cat#130-096-489).

### Experimental procedure

**Figure 1:**
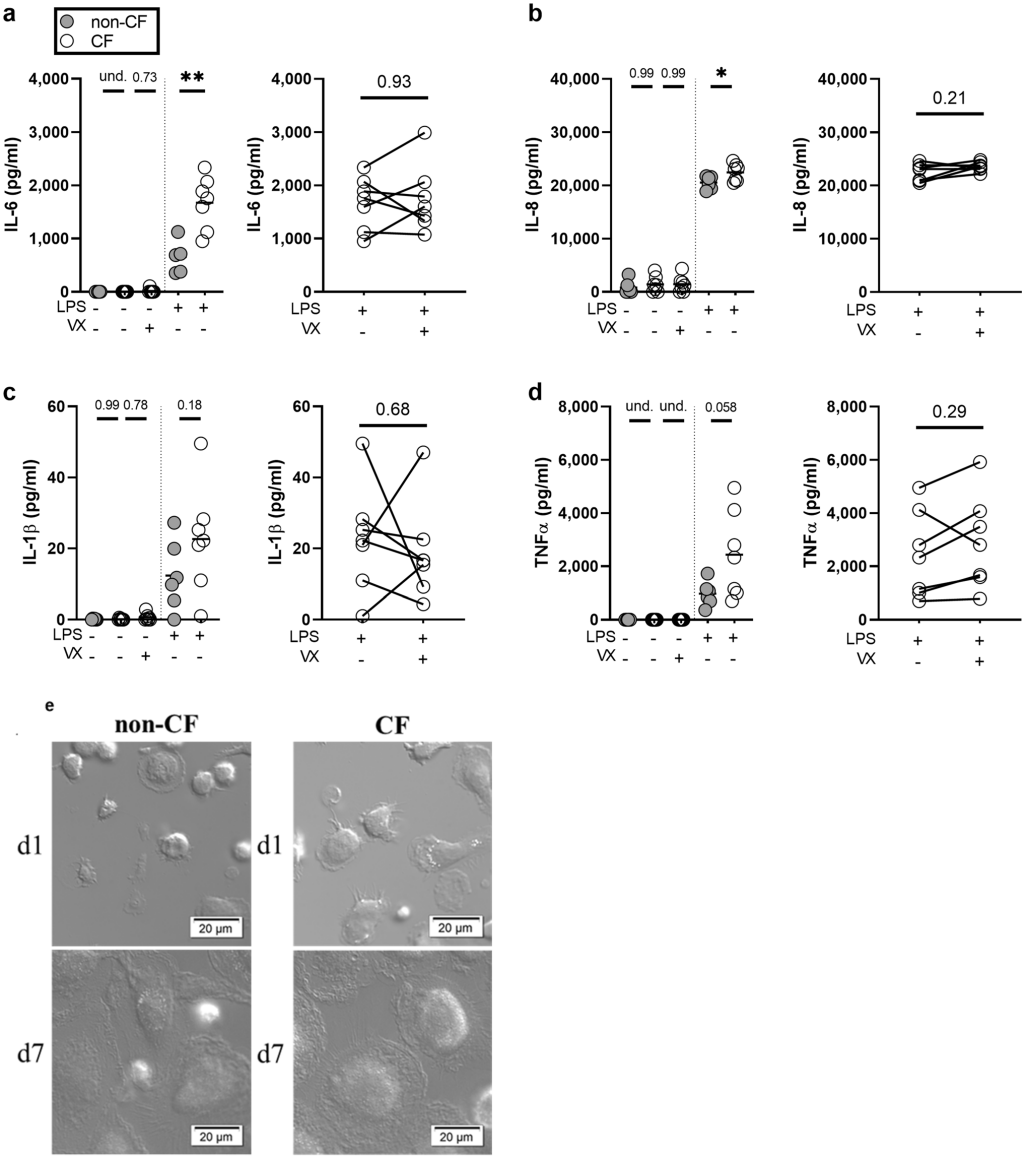
CF MDMs have a hyperinflammatory response to LPS. After 48 h pretreatment with vehicle or modulators (ivacaftor [30 nM] and lumacaftor [3 µM], VX), CF (n = 7) and non-CF (TNFα n = 5; IL-6, IL-8, and IL-1β n = 6) MDMs were exposed to vehicle or LPS for 24 h before supernatants were collected for ELISA for (**a**) IL-6, (**b**) IL-8, (**c**) IL-1β, and (**d**) TNFα. (‘*’ indicates P < 0.05, ‘**’ indicates P < 0.01). No-LPS controls were analyzed using non-parametric Kruskal–Wallis test with Dunn’s test for multiple comparisons. LPS-treated non-CF and CF groups were compared using unpaired t-tests. LPS-treated CF and LPS-treated CF with modulator pretreatment were compared using paired Wilcoxon rank sum tests. (**e**) Representative DIC microscopy images of non-CF (n = 2) and CF (n = 3) monocytes after differentiation with M-CSF for 1 and 7 days (d1, d7).

**Figure 2:**
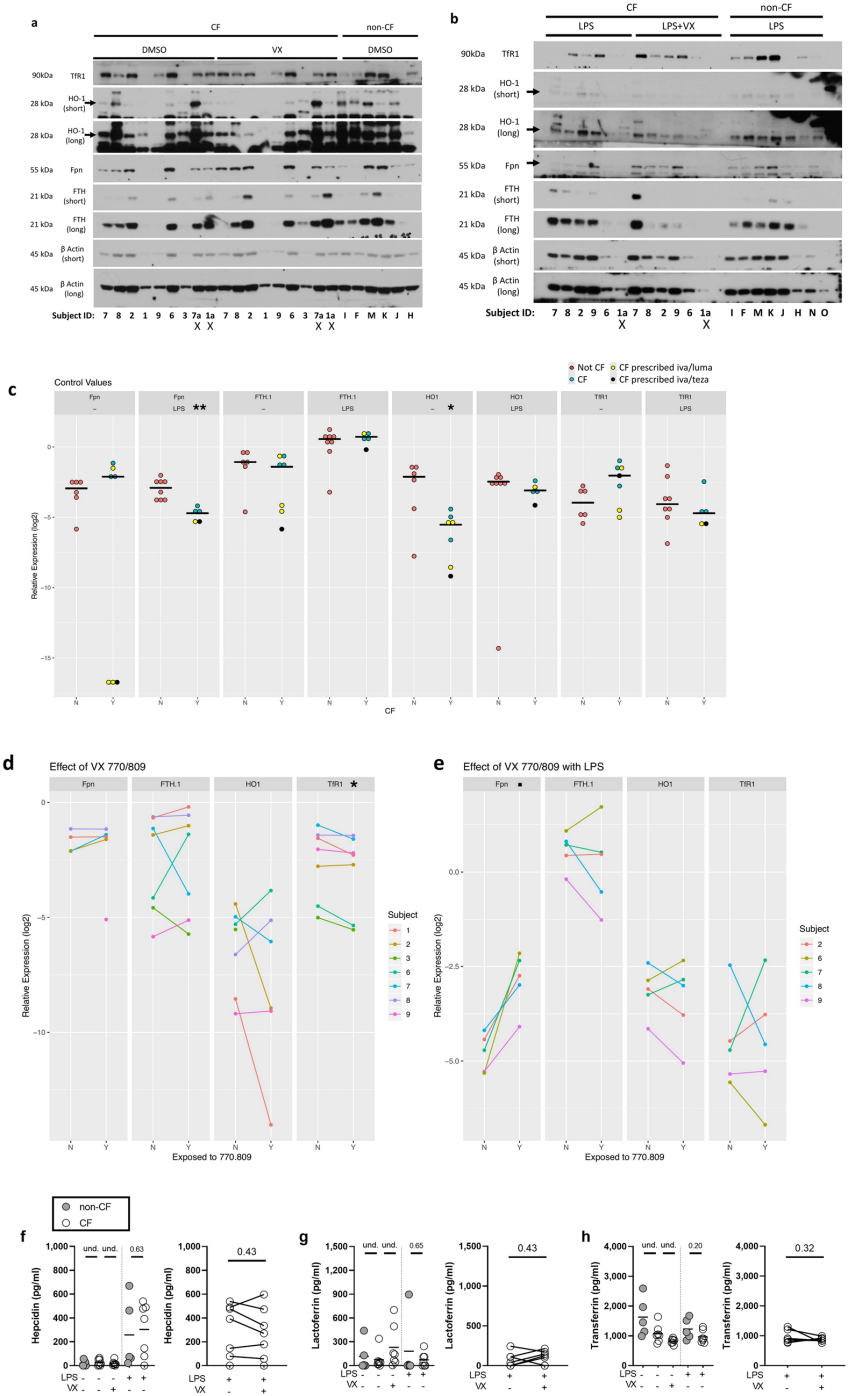
CF MDMs have a different iron-related protein expression profile compared to non-CF MDMs. Densitometry calculations were performed on samples run on the same membrane, at the same exposure time for each protein. Short and long exposure times are depicted for HO-1, FTH1, and β Actin. Numbers and letters denote arbitrary subject identification. “X” indicates the lane was excluded because this subject was a repeat donor. Black arrows indicate molecular weight of the protein in cases where there are multiple bands. Blots were cropped for presentation. Full images of blots are in Supplementary Fig. S5a and b. (**a**) Representative images of western blots in CF (n = 7) pretreated with vehicle or modulators and non-CF (n = 6) MDMs pretreated with vehicle, followed by exposure to vehicle for 18 h. (**b**) Representative images of western blots in CF (n = 8) and non-CF (n = 5) MDMs pretreated with vehicle or modulators, followed by LPS for 18 h. (**c–e**) After log2 transformation of the densitometric values, protein was normalized to β Actin and analyzed. Graphs were generated with R version 3.6.0 (2019-04-26, https://www.R-project.org/) (**c**) Baseline differences between CF and non-CF protein expression and differences between LPS-treated CF and non-CF protein expression were analyzed using Wilcoxon rank sum tests (‘*’ indicates P < 0.05, ‘**’ indicates P < 0.01). Within the CF population, whether a patient was using in vivo ivacaftor/lumacaftor or ivacaftor/tezacaftor was denoted by color (yellow “iva/luma” and black “iva/teza”). (**d**) The impact of modulators (ivacaftor [30 nM] and lumacaftor [3 µM], VX) on CF MDM protein expression was analyzed using linear models with factors to account for exposure and subject (‘*’ indicates P < 0.05). (**e**) The impact of modulators (ivacaftor [30 nM] and lumacaftor [3 µM], VX) on CF MDM protein expression in the presence of LPS was analyzed using linear models (‘.’ indicates P = 0.07). (**f**) Hepcidin, (**g**) lactoferrin, and (**h**) transferrin ELISAs were performed on supernatants collected from CF (n = 7) and non-CF (n = 5) MDMs pretreated with vehicle or modulators, followed by exposure to vehicle or LPS for 24 h. No-LPS controls were analyzed using non-parametric Kruskal–Wallis test with Dunn’s test for multiple comparisons. LPS-treated non-CF and CF groups were compared using Mann–Whitney tests. LPS-treated CF and LPS-treated CF with modulator pretreatment were compared using paired Wilcoxon rank sum tests.

**Figure 3:**
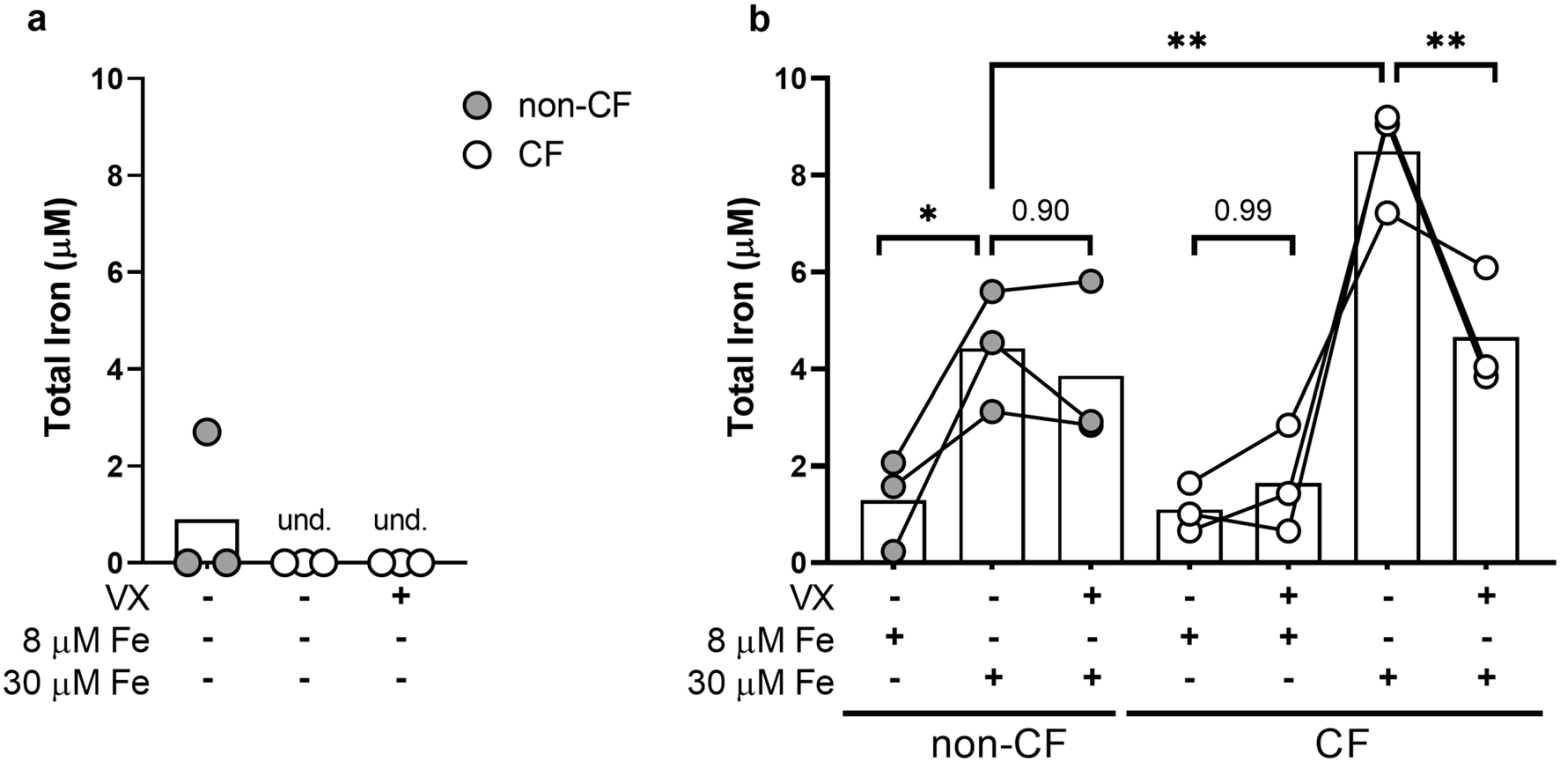
Non-CF MDMs deplete more exogenous iron than CF MDMs*.* Total iron (Fe^3+^/Fe^2+^) was measured in conditioned media from CF (n = 3) and non-CF (n = 3) MDMs after 48-h pretreatment in vehicle (DMSO) or modulators (ivacaftor [30 nM] and lumacaftor [3 µM], VX) followed by 48-h treatment with FeCl_3_. (**a**) Total iron measured in conditioned supernatants without the addition of exogenous iron. One-way ANOVA. Und. = undetectable. (**b**) Total iron measured in conditioned supernatants with the addition of exogenous iron. (‘*’ indicates P < 0.05, ‘**’ indicates P < 0.01, ‘***’ indicates P < 0.001, one-way ANOVA).

**Figure 4:**
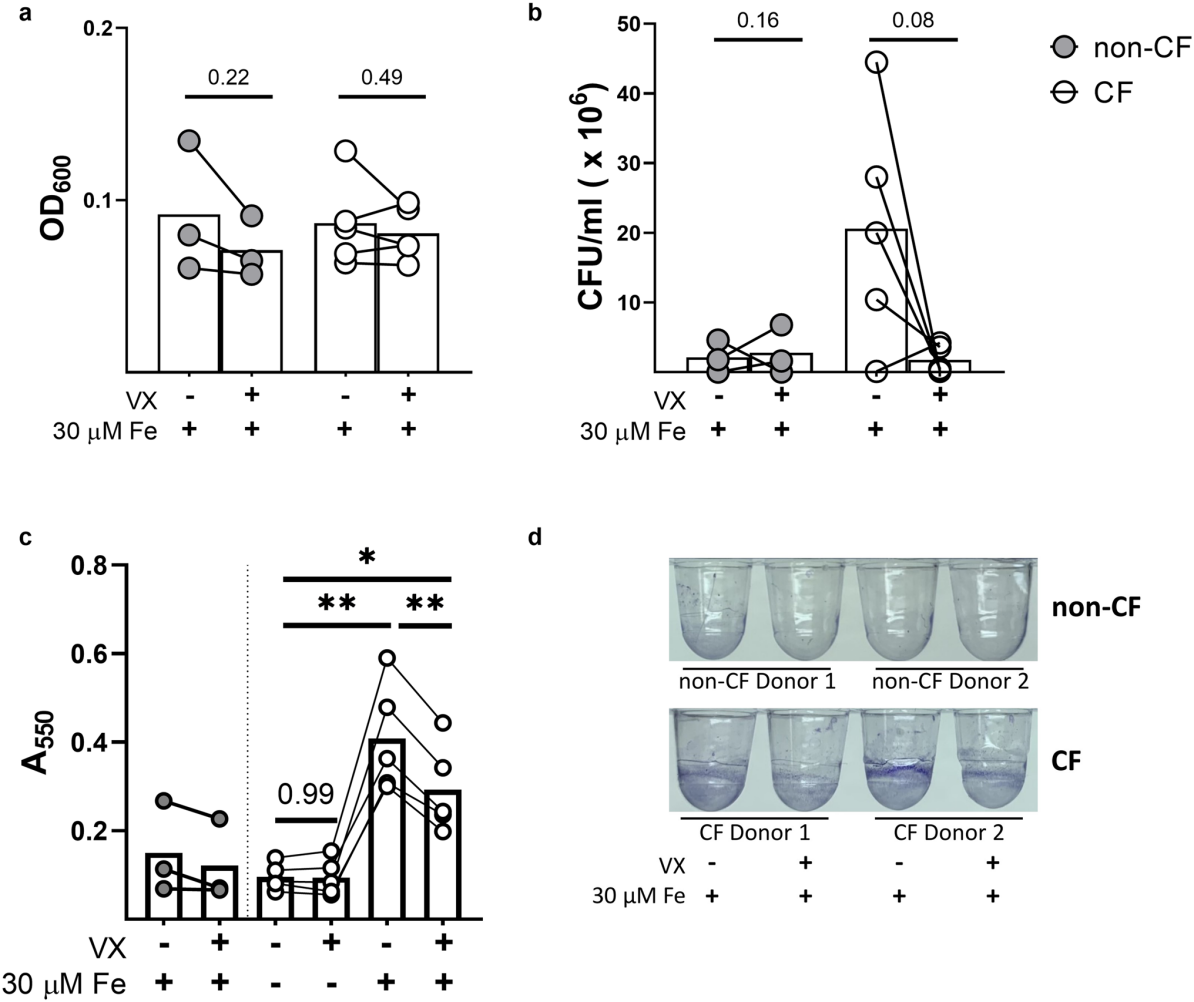
CF MDM conditioned media contributes to *Pa* biofilm formation and viability*.* CF (n = 5) and non-CF (n = 3) MDMs were pretreated for 48 h with vehicle (DMSO) or modulators (ivacaftor [30 nM] and lumacaftor [3 µM], VX). Conditioned media was collected after an additional 48 h incubation with 30 µM FeCl_3_. Bacterial biofilm formation and colony forming units (CFUs) were determined after 6 h exposure to conditioned media treated with 30 µM FeCl_3_. (**a**) Absorbance at λ = 600 nm optical density of the biofilm assay supernatant, analyzed with paired t-tests. (**b**) CFUs recovered from the biofilm assay supernatant, analyzed with paired t-tests. (**c**) Biofilm formation measured after exposure to conditioned media by measuring absorbance at λ = 550 nm. (‘*’ indicates P < 0.05, ‘**’ indicates P < 0.01, repeated measures one-way ANOVA). (**d**) Representative image of biofilm formation.

Experiments were performed in RPMI 1640 Glutamax with 50 µg/ml gentamicin with 10% HI-FBS. MDMs underwent 48-h pretreatment with 0.2% DMSO or combination 30 nM ivacaftor and 3 µM lumacaftor (modulators, Selleckchem, cat#S7059 and S1565). Modulator concentrations were based on CF subject drug plasma concentrations and those used in previously published works^27,52,53^. For lipopolysaccharide (LPS) and iron experiments, cells were washed after pretreatment and given 1 µg/ml *E. coli* O111:B4 LPS (Sigma, cat#L3024) for 24 h or 8–30 µM FeCl_3_ (Sigma, cat#157740) for 48 h, after which time conditioned media was collected and centrifugated 10 min, 13,000×*g*, 4 °C. Concentrations of FeCl_3_ were chosen to reflect a range of physiologically relevant iron levels observed in the CF airway: bronchoalveolar lavage fluid (mean ~ 25 ug/dL iron or ~ 4.4 µM iron in 10 CF children) or sputum (mean 35.3 µM in 6 CF adults during acute exacerbation)^15,16^. Vehicle or modulators were maintained in medium throughout as the half-life of lumacaftor is reported at approximately 18 h^54^ and the half-life of ivacaftor is reported as approximately 12 h by the 2012 “European Medicines Agency Committee for Medicinal Products for Human Use assessment report on ivacaftor”. Subjects were recruited for this study over a period of 2 years. Three CF subjects donated blood multiple times over the course of the study and are referred to as “repeat donors” in the text. Not all subjects yielded sufficient cells for all the experimental conditions listed in the manuscript, resulting in variable subject numbers for Figs. 1a–d and 2a–h. For Fig. 3a,b, experiments were performed on newly recruited or repeat donor macrophages (n = 3 per group). For Fig. 4a–d, experiments were performed on supernatant collected from the non-CF cohort in Fig. 3a,b. For Fig. 4a–d, due to supernatant availability, experiments were performed on supernatants from 2 of the CF cohort in Fig. 3a,b and on supernatants collected from 3 CF repeat donors.

### Biofilm formation and CFU quantification

*Pa* clinical isolate strain DH1137 (a generous gift from Dr. Deborah Hogan, previously described as strain NC-AMT0101-2^55^) was cultured in LB broth shaking overnight at 37 °C. Stationary-phase cultures were diluted 1:10 in 0.2% casamino acids, 0.4% l-arginine, 0.2% d-glucose, and 0.02% MgSO_4_ supplemented M63 biofilm formation media (US Biological, cat#M1015). Bacteria were then cultured to optical density (OD) at λ = 600 nm equal to 0.05 (1 × 10^8^ cells/ml). In vinyl 96-well plates (ThermoFisher Scientific, cat#2101), 75 µl DH1137 was diluted with 25 µl MDM conditioned media and incubated with humidity for 6 h at 37 °C. After incubation, 50 µl supernatant from each well was collected and OD at λ = 600 nm (total bacterial density) was measured in a microplate reader. Additionally, supernatant from each well was diluted 1:1,000 in LB broth and streaked onto agar plates for quantification of viable bacterial colony forming units (CFUs). Biofilm assay plates were washed with water, stained with 125 µl/well 0.1% crystal violet for 10 min, washed, and air dried. Photographs of biofilms were taken prior their quantification. After solubilizing biofilms with 150 µl/well 30% glacial acetic acid in water, absorbance at λ = 550 nm was used to quantitate each sample. Biofilm photographs were cropped (Fig. 4d), photographs of all subjects and conditions tested are available in Supplementary Fig. S6, metrics such as contrast and brightness were not altered for publication.

### Immunoblots

Total protein lysates were obtained using 200 µl/well 2 × Laemmli buffer (Bio-Rad, cat#1610737) with 5% β-mercaptoethanol with 1% protease inhibitor (ThermoFisher, cat#78425) and boiling 97 °C, 5 min. Lysates were run on 10% gels (Bio-Rad, cat#5671024, 5671034) and transferred to polyvinylidene difluoride (PVDF) membranes. Membranes were cut and incubated overnight, 4 °C, in 5% non-fat dried milk in TBS with 0.1% Tween20 (Carnation brand, NFDM, TBST) containing 1:5,000 primary: polyclonal rabbit anti-ferroportin (Novus, cat#NBP1-21502); monoclonal mouse anti-β Actin (abcam, cat#ab8226); monoclonal rabbit anti-heme oxygenase 1 (abcam, cat#ab68477); monoclonal mouse anti-transferrin receptor 1 (Santa Cruz, cat#sc-3227). Membranes were incubated for 1 h, room temperature, with 1:5,000 NFDM TBST mouse IgGκ binding protein (Santa Cruz, cat#sc-516102) or polyclonal goat anti-rabbit IgG (abcam, cat#ab7090) secondary antibodies. After development with Chemiluminescent Substrate (ThermoFisher, cat#34,580). Immunoblots depicted in Fig. 2 were scanned and converted to 8-bit images in ImageJ in order to quantify densitometry in ImageStudioLite v5.2. Different exposure times/blots were not compared to each other. For clarity of presentation, immunoblots in Fig. 2a,b were cropped and rearranged with a margin of white space around the cropped blots to denote that the blots were cropped. Full images of immunoblots prior to 8-bit conversion are in Supplementary Fig. S5a,b.

### ELISA

Supernatants were centrifugated 10 min, 13,000×*g*, 4 °C. ELISAs were performed according to manufacturer’s instruction: human Hepcidin (R&D Systems, cat#DY8307-05), IL-6 (R&D Systems, cat#DY602), TNFα (R&D Systems, cat#DY2010), IL-8 (R&D Systems, cat#DY208), lactoferrin (abcam, cat#ab108882), and transferrin (abcam, cat#ab108902).

### Cytotoxicity

Lactate dehydrogenase (LDH) cytotoxicity assay (ThermoFisher, cat#88,953) was performed on supernatants after centrifugation.

### Microscopy

Differential interference contrast (DIC) images were taken using an Olympus IX83 live cell microscope of monocytes seeded on glass-bottomed culture dishes.

### Statistical analysis

Data were analyzed using GraphPad Prism version 8.0.1. Subject sex distribution was analyzed with Fisher’s exact test. Data were analyzed where appropriate using unpaired two-tailed t-test and one-way ANOVAs with Tukey’s post-hoc test. Normality tests Anderson–Darling, D’Agostino–Pearson, Shapiro–Wilk, and Kolmogorov–Smirnov were performed automatically by GraphPad Prism with every analysis to determine whether data fit a Gaussian distribution. If data failed to pass a single normality test, nonparametric statistical tests were employed. The nonparametric test used for comparing two unpaired groups was the Mann–Whitney test, the Wilcoxon test was used for paired analysis within CF subjects, and the nonparametric test used for comparing more than two groups was the Kruskal–Wallis test with Dunn’s test for multiple comparisons. All figures except Fig. 3a–d (all male CF subjects) show data from male and female subjects. The lysis buffer employed in this study does not allow for protein quantification. Although monocytes were seeded equally at 5 × 10^5^ cells/well in 12-well culture plates, platelet contamination from the isolation procedure occasionally resulted in a crowding out of the monocytes and a reduced confluence of cells in some wells. We believe this to be the cause of the lower levels of β Actin in certain lanes of the western blots presented in Fig. 2a,b and Supplementary Fig. S5a, b. Densitometry results (Supplementary file 1) were imported into the R statistical programming environment (R version 3.6.0 [2019-04-26] https://www.R-project.org/) with an R script (Supplementary file 2). Briefly, zero values and values that were missing due to high background on the blot were removed. Rare instances where the same patient was measured multiple times were not included in this analysis to simplify the analysis. Ratios for proteins of interest were made to β Actin values in the same lane and log base 2 transformed to improve visualization and achieve normality. Graphs depicting comparisons between CF and non-CF MDMs as well as responses to ivacaftor/lumacaftor were generated using the R package ggplot2_3.2^56^ (https://CRAN.R-project.org/package=gplots, Fig. 2c–e). Statistically significant differences between CF and non-CF MDMs not exposed to ivacaftor/lumacaftor were assessed using Wilcoxon rank sum tests, a non-parametric alternative to t tests. Statistically significant differences between CF MDMs exposed to ivacaftor/lumacaftor were assessed by linear models that included factors for CF subject and exposure to ivacaftor/lumacaftor. In Supplementary Fig. S4a, b, heatmaps were produced with the R package gplots^57^ with the heatmap.2 function (gplots_3.0.1.2).

## Results

### Subject characteristics

Subject characteristics are summarized in Table 1. Ten cystic fibrosis (CF) subjects homozygous for the F508del CFTR mutation participated in this study. Twelve non-CF subjects participated in this study. Five of the ten CF subjects were taking CFTR modulators at the time of phlebotomy, three subjects were taking combination ivacaftor/lumacaftor, one subject was taking combination ivacaftor/tezacaftor, and one subject was taking combination ivacaftor/tezacaftor/elexacaftor. The use of clinical modulators was considered “in vivo” modulator treatment. Because we began our ex vivo studies on CF MDMs at a time when lumacaftor/ivacaftor was the only FDA approved modulator for F508del homozygous patients, this study was limited to the use of ex vivo pretreatment with lumacaftor/ivacaftor for experiments.

**Table 1:** Donor characteristics.

Characteristic	CF (n = 10)	Non-CF (n = 12)	P value
Sex, female % (n)	30% (3)	42% (5)	0.67
**Average age, years **(**range**)	28 (19–31)	25 (21–28)	0.61
Age (female)	30 (29–30)	24 (21–28)	0.24
Age (male)	27 (19–31)	26 (23–28)	0.99
Average FEV1, percent predicted (range)	78.2% (28–115%)		
**Sputum microbiology %**			
Infected with at least one bacterial species	60%		
Pa non-mucoid	40%		
Pa mucoid	40%		
Pa non-mucoid and mucoid	30%		
MSSA	40%		
MRSA	0%		
**CFTR therapeutics % **(**average FEV1%**)			
lumacaftor (VX-809) + ivacaftor (VX-770)	30% (83%)		
tezacaftor (VX-661) + ivacaftor (VX-770)	10% (28%)		
tezacaftor (VX-661) + ivacaftor (VX-770) + elexacaftor (VX-445)	10% (59%)		

Values are means (min–max range).

*n* no. of subjects, *FEV1* forced expiratory volume in 1s, *Pa Pseudomonas aeruginosa*, *MSSA* methicillin susceptible *Staphylococcus aureus*, *MRSA* methicillin resistant *Staphylococcus aureus.*

P values determined by Fisher’s exact test and one-way ANOVA.

### CF MDMs have a hyperinflammatory response to LPS

CF macrophages are well documented to express a hyper-inflammatory phenotype in response to bacterial stimuli including LPS and *Burkolderia cenocepacia*^46,50^. Using LPS to induce NFκB signaling and macrophage inflammatory activation, we measured several key cytokines that play an important role in inflammatory signaling and neutrophil recruitment in the CF airway. Compared to non-CF, we found that CF macrophages secreted significantly more IL-6 (P = 0.0102) and IL-8 (P = 0.0382) in response to 24 h exposure to LPS (Fig. 1a,b), but not IL-1β (Fig. 1c) or tumor necrosis factor alpha (TNFα) (Fig. 1d). Pretreatment for 48 h with modulators did not alter CF MDM cytokine secretion (Fig. 1a–d, pairwise comparisons within subject). We did not observe gross phenotypic differences in monocytes and MDMs over the course of differentiation (Fig. 1e, Supplementary Fig. S1), or cytotoxicity in response to treatment conditions as measured by lactate dehydrogenase (LDH) release at 24 h (Supplementary Fig. S2).

Four of the seven CF subjects whose macrophages were used for cytokine studies were taking prescribed CFTR modulator therapy at the time of phlebotomy [lumacaftor/ivacaftor (n = 3) or tezacaftor/ivacaftor (n = 1)]. Within this subset of our cohort, we found no differences in IL-1β, IL-6, or IL-8 secretion between CF subjects who were taking prescribed modulators and CF subjects who were not (Supplementary Fig. S3a–c). Compared to CF patients not on modulators in vivo, TNFα secretion was either trending towards or significantly (P = 0.0080) reduced regardless of pretreatment with modulators (Supplementary Fig. S3d). While the presumable in vivo exposure of monocytes to modulators in circulation may have lowered TNFα secretion (Supplementary Fig. S3d), ex vivo pretreatment with modulators for 48 h prior to LPS stimulation did not significantly alter cytokine secretion between the two CF subpopulations (Fig. 1a–d).

### CF MDMs have a different iron-related protein expression profile compared to non-CF MDMs

Iron metabolism is dysregulated in CFBEs, and macrophages found in CF lung explants contain elevated intracellular iron^13,16,17^. To determine if CF MDMs had differential iron-related protein levels, MDMs were assayed for soluble regulators of iron sequestration^21,38,39,58^: lactoferrin and transferrin, and the ferroportin (Fpn) regulatory hormone hepcidin. We also measured total protein expression of key iron handling proteins: the iron importer transferrin receptor 1 (TfR1), the iron exporter Fpn, the iron-storage protein ferritin (FTH1), and the anti-inflammatory heme catabolism enzyme, heme oxygenase 1 (HO-1). Comparing protein expression under control conditions between non-CF and CF, we found a trend towards elevated TfR1 (P = 0.1014) in CF MDMs (Fig. 2a,c). Compared to non-CF, CF HO-1 expression was lower (Fig. 2a,c), in agreement with previously published work^46^. No changes were observed in Fpn or FTH1 (Fig. 2a,c). Using pairwise comparisons to examine the impact of modulators on protein expression within subject, we found that modulator treatment reduced TfR1 levels (Fig. 2a,d). When activated with LPS, we found that CF macrophages expressed less Fpn and trended towards less HO-1 compared to LPS-activated non-CF macrophages (Fig. 2b,c). In the presence of LPS, ex vivo modulators induced a trend (P = 0.07) towards increased Fpn compared to LPS-activated non-CF macrophages (Fig. 2b,e). Across treatment groups, soluble regulators of iron sequestration and hepcidin (Fig. 2f–h) showed no differences between CF and non-CF MDMs. Regardless of ex vivo modulator pretreatment, we found that prescribed modulator use in vivo coincided with a trend in lower hepcidin secretion in LPS-activated CF MDMs compared to LPS-activated CF MDMs from patients who did not use in vivo modulators (Supplementary Fig. S3e). Compared to LPS-activated non-CF MDMs, we found that in vitro modulator-pretreatment in LPS-activated CF MDMs from patients on prescribed modulators had elevated lactoferrin secretion (P = 0.0618, Supplementary Fig. S3f), and significantly lower transferrin (P = 0.0076, Supplementary Fig. S3g). Clinical use of modulators variably impacted expression of Fpn, FTH1, HO-1, and TfR1 (Supplementary Fig. S4a,b). These observations demonstrate that CF may influence expression of certain iron-regulatory proteins, and that both ex vivo modulator pretreatment and in vivo use of prescribed modulators have the potential to differentially influence soluble iron-regulatory protein expression.

### Non-CF MDMs deplete more exogenous iron than CF MDMs

Lung damage leads to elevated levels of red blood cells, heme, and iron in the CF airway^16^. Mean concentrations of iron ranging from 4 to 35 µM have been measured in CF airways and sputum^15,16^. The ability of macrophages to sequester iron is a key component in preventing both oxidative tissue damage and bacterial iron metabolism. Having found that CF MDMs expressed altered TfR1 and Fpn protein levels (Fig. 2c), both of which are critical for management of iron in the microenvironment, we sought to focus on CF MDM iron handling by examining total iron content of conditioned media after the addition of clinically relevant concentrations of iron^15,16^. Given that recruited macrophages would be chronically exposed to high levels of iron in the CF airway, we hypothesized that CF MDMs challenged with physiologically relevant concentrations of iron would not sequester and/or retain exogenous iron as effectively as non-CF MDMs. As a surrogate for macrophage iron uptake or sequestration, we measured the depletion of exogenously added iron from the culture medium after 48 h.

In control groups that did not receive exogenous iron, the concentration of total iron was undetectable by our colorimetric assay (Fig. 3a). We found that 8 µM FeCl_3_ was cleared to levels below the assay detection range by both non-CF and CF MDMs (Fig. 3b). At a higher concentration of exogenous iron (30 µM), conditioned media from CF MDMs contained significantly (P = 0.0044) higher levels of iron than non-CF (Fig. 3b). Additionally, 48-h pretreatment with CFTR modulators ameliorated this effect in CF MDMs (Fig. 3b). These findings imply that CF MDMs sequester and/or withhold less iron than non-CF.

### CF MDM conditioned media contributes to Pa biofilm formation and viability

We next hypothesized that elevated iron levels in CF MDM conditioned media may influence the growth of a CF *Pa* clinical isolate, DH1137. To do this, we collected supernatants from non-CF and CF MDMs that were given exogenous iron, with or without modulator pretreatment (Fig. 3b). We chose to test supernatants from MDMs exposed to 30 µM FeCl_3_ because we observed significant and measurable changes in the amount of iron present in CF and modulator-pretreated CF MDM supernatants at this concentration (Fig. 3b). We mixed the MDM supernatants with *Pa* DH1137 and performed a biofilm formation assay. At the endpoint of the biofilm formation assay, we recovered the mixed supernatants to measure *Pa* density and viability. We found that the optical density (OD) at λ = 600 nm (OD_600_) of the supernatant recovered from the biofilm formation assay was not altered (Fig. 4a), indicating no change in the density of bacteria in the assay supernatant. However, the OD_600_ reading does not discern the relative density of live, dead, or dying bacteria. To answer this question, we measured viable bacteria recovered from the biofilm formation assay by measuring the growth of colony forming units (CFUs) recovered from the assay supernatant. We found that CF conditioned media increased recovery of viable bacteria (Fig. 4b, mean: non-CF viable CFU × 10^6^ = 2.133 ± standard deviation (SD) 2.30, CF viable CFU × 10^6^ = 20.60 ± SD 16.9). Additionally, CF conditioned media increased the formation of biofilms, as measured by absorbance at λ = 550 nm (A_550_) (Supplementary Fig. S6, Fig. 4c,d, mean: non-CF biofilm formation A_550_ = 0.150 ± SD 0.104, CF biofilm formation A_550_ = 0.408 ± SD 0.124). Modulator-pretreated CF conditioned media reduced the recovery of viable bacteria (P = 0.0876, Fig. 4b) and significantly reduced biofilm formation (P = 0.0033, Fig. 4c,d).

## Discussion

While iron may not be readily available in the non-CF human lung, both CF sputum and airway lavage fluid contain elevated iron^15,16,59^. Iron is an essential nutrient for the antibiotic resistance and biofilm formation of *Pa*, one of the most common CF pathogens^13,60,61^. As manipulation of iron availability has become a therapeutic option of interest for CF lung infections^60–62^, expanding our understanding of iron metabolism in primary CF macrophages could offer insight into airway host–pathogen interactions.

The goal of this study was to test the hypotheses that (1) iron-regulatory proteins are altered in CF MDMs, and (2) ex vivo ivacaftor/lumacaftor corrects expression of iron-regulatory proteins in a manner relevant to host–pathogen interactions. We analyzed samples obtained from a small cohort of CF patients who all had a common and severe CFTR mutation (Class II, F508del/F508del). We exposed primary human MDMs to physiologically relevant concentrations of CFTR modulators^27,53,63^ and iron^15,16^, and examined cytokines, iron-carrier proteins, the hormone hepcidin, and cell-expressed proteins. We tested the impact of macrophage conditioned media on biofilm formation and viability of a *Pa* CF clinical isolate.

We found that CF MDMs produced more IL-6 and IL-8 (Fig. 1a,b) and less of the anti-inflammatory heme catabolism enzyme, HO-1 (Fig. 2c). These findings are in support of extensive works of Bruscia et al. on the characterization of CFTR-dependent HO-1 dysregulation in both murine and human macrophages and the contribution of this dysregulation to CF macrophage hyperinflammation^46,47^. In the context of infection and inflammation, the transcription of the Fpn-regulatory hormone hepcidin is induced by IL-6-activated JAK/STAT signaling^38^. While CF MDMs secreted significantly more IL-6 than non-CF MDMs (Fig. 1a, P = 0.0102), there was no difference in hepcidin secretion (Fig. 2f). Though we observed significantly lower Fpn in CF MDMs with LPS activation (P = 0.0015, Fig. 2c), we did not observe changes in hepcidin secretion under these conditions (Fig. 2f). While modulator treatment ex vivo did not impact hepcidin secretion, we did observe trends in reduced hepcidin secretion in subjects taking modulators in vivo (Supplementary Fig. S3e). Though in vivo modulator use induced trends in reduced hepcidin secretion (Supplementary Fig. S3e), we found no increase in Fpn expression with in vivo modulator use (Supplementary Fig. S4a,b), suggesting the observed reduction in hepcidin secretion was not responsible for appreciable changes in Fpn expression. Although we found that in vivo modulator use coincided with significantly lower transferrin (P = 0.0076) in activated, modulator-pretreated CF MDMs (Supplementary Fig. S3g), TfR1 protein expression was not altered in association with patient modulator use under any treatment conditions (Supplementary Fig. S4a,b). These findings demonstrate that clinical use of modulators but not ex vivo modulator treatment can impact inflammatory and iron-related protein secretion (Supplementary Fig. S3d–g). This suggests that acute and chronic modulator exposure differentially impacts monocyte/macrophage iron handling and immunophenotype. This may be addressed in future studies of patients taking the same triple combination therapy.

In addition to lower Fpn in CF MDMs with LPS activation (Fig. 2c), we found that ex vivo modulator pretreatment in LPS-activated MDMs induced a trend towards elevated Fpn without altering IL-6 or hepcidin secretion (Figs. 1a, 2c,f, Supplementary Fig. S3e). While it is known that hepatocytes are the primary producers of hepcidin^64^, these findings pose interesting questions about hepcidin-independent mechanisms of Fpn regulation as well as potential autocrine dysregulation of the hepcidin-ferroportin regulatory axis. These findings also offer insight into iron-withholding in CF MDMs activated through TLR4. CF MDMs have a proven deficit to phagocytose and kill *Pa*^27^, suggesting that lower Fpn in activated CF MDMs does not impart an advantage to the host during direct host–pathogen interactions. Fpn transports iron (II) across the cell membrane and releases iron (II) to be oxidized and finally bound to transferrin as iron (III)^65^. We observed no change in FTH (Fig. 2c), which plays an important role preventing iron (II)-induced oxidative damage by oxidizing and storing iron (II) as iron (III)^66^. Although we did not measure intracellular iron content in this study, we can hypothesize that low CF MDM HO-1 and Fpn in conjunction with unchanged ferritin levels may contribute to elevated intracellular iron and oxidative stress (Fig. 2c). Elevated intracellular iron has been described in CFBEs and alveolar macrophages from CF lung explants, though alveolar macrophages in CF lung explants did also express elevated ferritin and ferroportin staining^13,16^. These questions may be addressed in future studies by measuring activated CF macrophage ROS production and intracellular iron content in response to exogenous iron.

CF MDMs expressed a trend towards elevated basal TfR1 (Fig. 2c) compared to non-CF MDMs, and basal TfR1 levels were significantly (P = 0.0259) reduced with modulator pretreatment (Fig. 2d). These data are intriguing and seemingly contradictory to our finding of elevated extracellular iron in CF MDM conditioned media (Fig. 3b). There are several avenues to consider when interpreting these data. TfR1 protein accumulation, in the absence of iron uptake, may stem from defects in protein internalization. This was found to be the case in a study of PBMCs and lymphocytes from human patients with a mutation in TfR1 gene, *TFRC*, in which internalization of TfR1 was disrupted, resulting in protein accumulation on the cell surface^67^. TfR1 mRNA stability is regulated by the zinc-finger protein tristetraprolin (TTP), which is in turn regulated by the mammalian target of rapamycin complex 1 (mTORC1)^68^. mTORC1 expression is elevated in CFBEs^69^ and TTP deficiency has been linked to hypersecretion of IL-8 from IB3-1 CF lung epithelial cells^70^, providing an additional potential avenue for TfR1 upregulation in CF MDMs.

No differences were found in IL-1β secretion from non-CF and CF LPS-activated macrophages (Fig. 1c). These data suggest similar NLRP3 inflammasome activation, although further studies would need to be done to measure pro-IL-1β and caspase-1 to further characterize inflammasome activity in LPS-activated CF macrophages. Findings in the literature suggest that differences in CF inflammasome activity may be observed in a context-dependent manner: CF airway epithelial cells and CF monocytes have similar IL-1β and caspase-1 activity in response to LPS^71^, while CF MDMs challenged with *Burkholderia cenocepecia* secrete significantly more IL-1β than non-CF^50^, and CFTR knockout mice challenged intranasally with *Aspergillus fumigatus* conidia or *Pa* express elevated IL-1β in lung homogenates^72^.

Except for TNFα, we found that in vivo ivacaftor/lumacaftor and tezacaftor/ivacaftor use largely did not impact LPS induced cytokine secretion (Supplementary Fig. S3a–d). Ex vivo pretreatment with ivacaftor/lumacaftor reduced expression of TfR1 and induced a trend towards elevated Fpn expression (Fig. 2d,e). These findings are in support of a recent publication in which MDMs isolated from CF patients taking clinical ivacaftor had improved *B. cenocepecia* phagocytosis and CFTR expression compared to MDMs isolated from CF patients who were taking ivacaftor/lumacaftor^50^. In that study, the genotype of donors on ivacaftor was comprised of ivacaftor-responsive class III/IV/V CFTR mutations^50^. Indeed, recent findings suggest that the combination of ivacaftor/lumacaftor induces negative effects on phagocytosis in CF MDMs^27,50^. Although we did not observe a significant detriment to biofilm clearance resulting from ex vivo ivacaftor/lumacaftor treatment, our findings that ex vivo treatment with ivacaftor/lumacaftor did not recover cytokine or soluble protein levels in CF MDMs (Figs. 1a–d, 2f–h) further demonstrate that ivacaftor/lumacaftor may not rescue all aspects of CF macrophage function in patients with more severe mutations. Additionally, the ex vivo tissue culture environment lacks many components such as cytokines, chemokines, elevated iron content, and other cell types that may influence how modulator therapy impacts innate immune cell function in vivo. Our future studies involve investigating macrophage function with an emphasis on iron homeostasis in patients who have consistently taken highly effective modulator therapy for over one year. These studies will allow us to assess the impact of highly effective modulator therapy in vivo on macrophage iron homeostasis.

In addition to reducing basal TfR1 and elevating Fpn in activated CF MDMs, we observed that ex vivo pretreatment with modulators robustly impacted CF MDM iron sequestration and biofilm formation of DH1137 (Figs. 3b, 4c). Elevated extracellular iron and the associated increase in *Pa* biofilm formation and viability (Fig. 4b–d) suggest that dysfunctional iron metabolism in CF MDMs (1) impacts host–pathogen interactions and (2) may be sensitive to CFTR modulator therapy. These findings are particularly relevant in the context of the CF lung, in which iron content is positively correlated with *Pa* abundance and in which *Pa* biofilms have been found^10,15^. When considering these data, it is important to understand that these studies do not directly test how iron effects *Pa*, but rather how iron-rich CF conditioned media effects *Pa*. To help determine if there is a causative rather than an associative relationship between iron levels in CF media and biofilm formation, direct manipulation of iron levels in conditioned media would be necessary.

These data raise several questions that require future studies. The expression of Fpn and TfR1 over a more comprehensive time course and CFTR modulator dose response regime would allow for greater granularity of expression profiles and potentially answer how modulators correct iron sequestration. Peripheral blood monocytes are recruited to the lung during infection and thus, represent a relevant model in which to study CF mononuclear phagocyte function. However, it is important to note that the lung environment shapes macrophage phenotype^73^. Due to the unique niche they occupy, CF alveolar macrophages may express an iron-regulatory phenotype that is different from that of CF MDMs. The ontogeny and lifecycle of alveolar macrophages are distinct from peripheral blood MDMs in that they are long-lived, self-renewing, tissue-resident cells derived from fetal progenitors^74^. They play a critical role in regulating the immune responses of the lung, inducing tolerance to innocuous inhaled antigens^75^ or triggering synchronous inflammatory signaling in response to pathogenic stimuli such as LPS^76^. Future studies on CF and non-CF alveolar macrophages would allow for greater clarity on potential mechanisms of dysregulated iron-handling in the airways themselves. Additionally, several studies have demonstrated the phenotypic impact of iron levels on inflammation, polarization, differentiation, and cellular metabolism^48,77–79^. We must also consider that elevated iron in the CF lung may impact the differentiation and phenotype of recruited monocytes as well as tissue resident macrophages, and thus modulate their iron-regulatory protein repertoire. Future studies in which CF macrophages are cultured in high-iron media from the time of monocyte isolation to macrophage differentiation may help answer these questions.

We have shown that CF MDMs are hyperinflammatory in phenotype and have altered expression of several iron related proteins that are altered with modulator treatment. CF MDMs have an inability to reduce levels of exogenous extracellular iron, and their iron-rich conditioned media directly facilitates bacterial biofilm formation and viability. We have shown that CFTR modulators correct aspects of dysfunctional iron metabolism in CF MDMs, although future studies are needed to further delineate the mechanisms of these results.

## Supplementary information

Supplementary file1 (XLSX 20 kb)

Supplementary file2 (DOCX 17 kb)

Supplementary file3 (PDF 1106 kb)
