# Supplementary material for: Altered iron metabolism in cystic fibrosis macrophages: the impact of CFTR modulators and implications for Pseudomonas aeruginosa survival
Source: Sci Rep. 2020 Jul 2;10:10935. doi: 10.1038/s41598-020-67729-5 (PMC7331733; doi:10.1038/s41598-020-67729-5)
Supplement: Supplementary file 2 — Supplementary file2 (DOCX 17 kb) [file 41598_2020_67729_MOESM2_ESM.docx]

library(readxl)

densitometry <- read_xlsx("Model_Format_AllData_SciRp2019.xlsx", sheet = 1 )

densitometry$`CF (Y/N)`[densitometry$`CF (Y/N)` == "non-CF"] <- "N"

densitometry$`CF (Y/N)`[densitometry$`CF (Y/N)` != "N"] <- "Y"

densitometry$`770/809 at time of phlebotomy (Y/N)`[ densitometry$`CF (Y/N)`=="N"] <- "N"

densitometry$`770/661 at time of phlebotomy (Y/N)` [ densitometry$`CF (Y/N)`=="N"] <- "N"

densitometry$`770/661/445 at time of phlebotomy (Y/N)` [densitometry$`CF (Y/N)`=="N"] <- "N"

densitometry$onVX <- apply(densitometry[, 3:5], 1, function (x){

ifelse("Y" %in% x, "Y", "N")

} )

dHasRef <- as.data.frame(densitometry[!is.na(densitometry$`Beta Actin Density` )

& !is.na(densitometry$`Blot #`) , ])

colnames(dHasRef) <- make.names(colnames(dHasRef))

write.csv(dHasRef, "dHasRef.csv")

summary(dHasRef[,c(10, 12, 14, 16)])

# set na to zero

dHasRef$HO.1.Density[is.na(dHasRef$HO.1.Density) ] <- 0

summary(dHasRef[,c(10, 12, 14, 16)])

apply(dHasRef[,c(10, 12, 14, 16)], 2, function (x){

sum(x == 0)

})

# TfR1.Density Fpn.Density HO.1.Density FTH.1.Density

# 0 5 2 0

# Set zero cells to 1 (exclude later)

dHasRef$Fpn.Density[dHasRef$Fpn.Density == 0] <- 1

dHasRef$HO.1.Density[dHasRef$HO.1.Density == 0] <- 1

# Make ratios to Beta Actin

dHasRef$TfR1 <- log2(dHasRef$TfR1.Density/dHasRef$Beta.Actin.Density)

dHasRef$Fpn <- log2(dHasRef$Fpn.Density/dHasRef$Beta.Actin.Density)

dHasRef$HO1 <- log2(dHasRef$HO.1.Density/dHasRef$Beta.Actin.Density )

dHasRef$FTH.1 <- log2(dHasRef$FTH.1.Density/dHasRef$Beta.Actin.Density )

# drop duplicate measurements of the same donor

dHasRefu <- dHasRef[grep ("a", dHasRef$Subject.ID , invert = TRUE),]

# Identify CF samples

DensRespCF <- dHasRefu[dHasRefu$CF..Y.N. == "Y" , ]

# Split CF samples by blot (+/- LPS)

DensRespCF1 <- DensRespCF[DensRespCF$Blot.. == "1",] # No LPS, blot 1

DensRespCF2 <- DensRespCF[DensRespCF$Blot.. == "2",] # LPS, blot 2

# Transform data to long formaT

library(tidyr)

colnames(DensRespCF1)[c(1,6,10,12,14,16, 18:22)]

DensRespCF1.l <- gather(DensRespCF1[, c(1,6,10,12,14,16, 18:22)], key = "Protein", value = "Exp",

-c("Subject.ID" , "X770.809..Y.N.",

"TfR1.Density", "Fpn.Density",

"HO.1.Density","FTH.1.Density", "onVX"))

colnames( DensRespCF1.l) <- c("ID", "VX", "TfR1.Density", "Fpn.Density", "HO1.Density", "FTH.1.Density", "onVX" ,

"Protein", "Exp")

# Identify which observations were artificially set to 1

Readings2Exclude <- unlist(lapply(unique(DensRespCF1.l$Protein) , function(p) {

which(DensRespCF1.l$Protein == p & DensRespCF1.l[,grep(p, colnames(DensRespCF1.l))] == 1)

}))

DensRespCF1.l[Readings2Exclude,] # all very tiny

# Exlude observations were artificially set to 1

DensRespCF1.l <- DensRespCF1.l[ -c(Readings2Exclude), ]

# âCF vs CF+770.809â

# Use a linear model to assess response to in vitro VX response, accounting for patient variability

CFvxModels <- lapply(unique(DensRespCF1.l$Protein), function(p){

summary(lm(Exp ~ VX + ID , data = DensRespCF1.l[DensRespCF1.l$Protein == p ,] ))

} )

names(CFvxModels) <- unique(DensRespCF1.l$Protein)

# Plot

library(ggplot2)

pdf("VXfacetCF.pdf")

ggplot(data = DensRespCF1.l, aes(x=VX, y=Exp, colour = ID, group = ID)) +

geom_point() + geom_line() +

labs(title="Effect of VX 770/809",

y="Relative Expression (log2)",

x="Exposed to 770.809",

color="Subject") +

facet_grid(cols = vars(Protein), scales = "free_y")

dev.off()

# âCF+LPS vs CF+LPS+770.809â

# Transform data to long format

DensRespCF2.l <- gather(DensRespCF2[, c(1,6,10,12,14,16, 18:22)], key = "Protein", value = "Exp",

-c("Subject.ID" , "X770.809..Y.N.",

"TfR1.Density", "Fpn.Density",

"HO.1.Density","FTH.1.Density", "onVX"))

colnames( DensRespCF2.l) <- c("ID", "VX", "TfR1.Density", "Fpn.Density", "HO.1.Density", "FTH.1.Density", "onVX" ,

"Protein", "Exp")

# No data to remove

# Use a linear model to assess response to in vitro VX response in LPS treated samples, accounting for patient variability

CFvxModelsLPS <- lapply(unique(DensRespCF2.l$Protein), function(p){

summary(lm(Exp ~ VX + ID , data = DensRespCF2.l[DensRespCF1.l$Protein == p ,] ))

} )

names(CFvxModelsLPS) <- unique(DensRespCF2.l$Protein)

pdf("VXfacetLPS.CF.pdf")

ggplot(data = DensRespCF2.l, aes(x=VX, y=Exp, colour = ID, group = ID)) +

geom_point() + geom_line() +

labs(title="Effect of VX 770/809 with LPS",

y="Relative Expression (log2)",

x="Exposed to 770.809",

color="Subject") +

facet_grid(cols =vars(Protein), scales = "free_y")

dev.off()

# ânon-CF vs CFâ and ânon-CF+LPS vs CF+LPSâ

Controls <- dHasRefu[dHasRefu$X770.809..Y.N. == "N" & dHasRefu$Blot.. %in% c("1", "2") , ]

colnames(Controls)[c(1,2,7, 19:22)]

Controls.l <- gather(Controls[, c(1,2,7, 19:22)], key = "Protein", value = "Exp",

-c("Subject.ID","CF..Y.N.", "LPS..Y.N."))

colnames(Controls.l) <- c("ID", "CF", "LPS", "Protein", "Exp")

Controls.l$LPS[Controls.l$LPS == "Y"] <- "LPS"

Controls.l$LPS[Controls.l$LPS == "N"] <- "-"

LPSwilcoxControls <- lapply(unique(Controls.l$Protein), function(p){

wilcox.test(Exp ~ CF, data = Controls.l[Controls.l$Protein == p & Controls.l$LPS == "LPS" ,])

} )

names(LPSwilcoxControls ) <- unique(Controls.l$Protein)

LPSmedianControls <- lapply(unique(Controls.l$Protein), function(p){

d = data.frame(Controls.l[Controls.l$Protein == p ,])

tapply(d$Exp, list( d$CF, d$LPS), median)

} )

names(LPSmedianControls ) <- unique(Controls.l$Protein)

noLPSwilcoxControls <- lapply(unique(Controls.l$Protein), function(p){

wilcox.test(Exp ~ CF, data = Controls.l[Controls.l$Protein == p & Controls.l$LPS == "-" ,])

} )

names(noLPSwilcoxControls ) <- unique(Controls.l$Protein)

Controls.l$LPS == "-"

pdf("ControlDots.pdf", width = 15)

ggplot(data = Controls.l, aes(x=CF, y=Exp, group = CF)) +

geom_dotplot(aes(fill = CF),

binaxis = "y", # which axis to bin along

binwidth = 0.3, # Minimal difference considered diffeerent

stackdir = "center" # Centered

) +

stat_summary(fun.y = median, fun.ymin = median, fun.ymax = median,

geom = "crossbar", width = 0.6) +

labs(title="Control Values",

y="Relative Expression (log2)",

x="CF") +

facet_grid(~ Protein + LPS, scales = "free_y")

dev.off()

pdf("ControlFacetLPS.CF.pdf", width = 10, height=6)

ggplot(data = Controls.l, aes(x=CF, y=Exp, group = CF)) +

geom_point() +

labs(title="Control Values",

y="Relative Expression (log2)",

x="CF") +

facet_grid(~ Protein + LPS, scales = "free_y")

dev.off()

library(gplots)

Controls$X770.809.at.time.of.phlebotomy..Y.N.

Controls$X770.661.at.time.of.phlebotomy..Y.N.

Controls$X770.661.445.at.time.of.phlebotomy..Y.N.

NoLPSControls <- Controls[Controls$LPS..Y.N. == "N", ]

NoLPSControls$Color <- "white"

NoLPSControls$Color [NoLPSControls$X770.809.at.time.of.phlebotomy..Y.N. == "Y"] <- "blue"

NoLPSControls$Color [NoLPSControls$X770.661.at.time.of.phlebotomy..Y.N. == "Y"] <- "green"

pdf("S4c.pdf")

heatmap.2(as.matrix(NoLPSControls[, 19:22] ),

labRow = paste(sapply(NoLPSControls$CF..Y.N., function(x){

ifelse(x == "Y", "CF:", "non-CF:")

}), NoLPSControls$Subject.ID),

scale = "column",

trace="none",

RowSideColors = NoLPSControls$Color,

margins = c(7,8),

main="Effect of 770.809")

dev.off()

LPSControls <- Controls[Controls$LPS..Y.N. == "Y", ]

LPSControls$Color <- "white"

LPSControls$Color [LPSControls$X770.809.at.time.of.phlebotomy..Y.N. == "Y"] <- "blue"

LPSControls$Color [LPSControls$X770.661.at.time.of.phlebotomy..Y.N. == "Y"] <- "green"

pdf("S4b.pdf")

heatmap.2(as.matrix(LPSControls[, 19:22] ),

labRow = paste(sapply(LPSControls$CF..Y.N., function(x){

ifelse(x == "Y", "CF:", "non-CF:")

}), LPSControls$Subject.ID),

scale = "column",

trace="none",

RowSideColors = LPSControls$Color,

margins = c(7,8),

main="Effect of 770.809, with LPS")

dev.off()
