# Supplementary material for: Altered iron metabolism in cystic fibrosis macrophages: the impact of CFTR modulators and implications for Pseudomonas aeruginosa survival
Source: Sci Rep. 2020 Jul 2;10:10935. doi: 10.1038/s41598-020-67729-5 (PMC7331733; doi:10.1038/s41598-020-67729-5)
Supplement: Supplementary file 3 — Supplementary file3 (PDF 1106 kb) [file 41598_2020_67729_MOESM3_ESM.pdf]

Supplementary Information for:

"Altered iron metabolism in cystic fibrosis macrophages: the impact of CFTR modulators and implications for *Pseudomonas aeruginosa* survival."

Hazlett, H.F.[2], Hampton, T.H.[2], Aridgides, D.S.[1], Armstrong, D.A.[1], Dessaint J.A.[1], Mellinger, D.L.[1], Nymon, A.B.[2], and *Ashare, A.[1,2]

[1]*Medicine, Dartmouth-Hitchcock Medical Center, Lebanon, NH, USA.* [2]*Microbiology and Immunology, Dartmouth College, Hanover, NH, USA.*

Correspondence (production): H. Hazlett, Dept. of Microbiology and Immunology, Geisel School of Medicine at Dartmouth. 1 Medical Center Dr, Lebanon, NH 03756.

Email: Haley.f.hazlett.gr@dartmouth.edu.

Correspondence: A. Ashare, Dept. of Medicine, Geisel School of Medicine at Dartmouth. 1 Medical Center Dr, Lebanon, NH 03756.

Email: Alix.Ashare@hitchcock.org

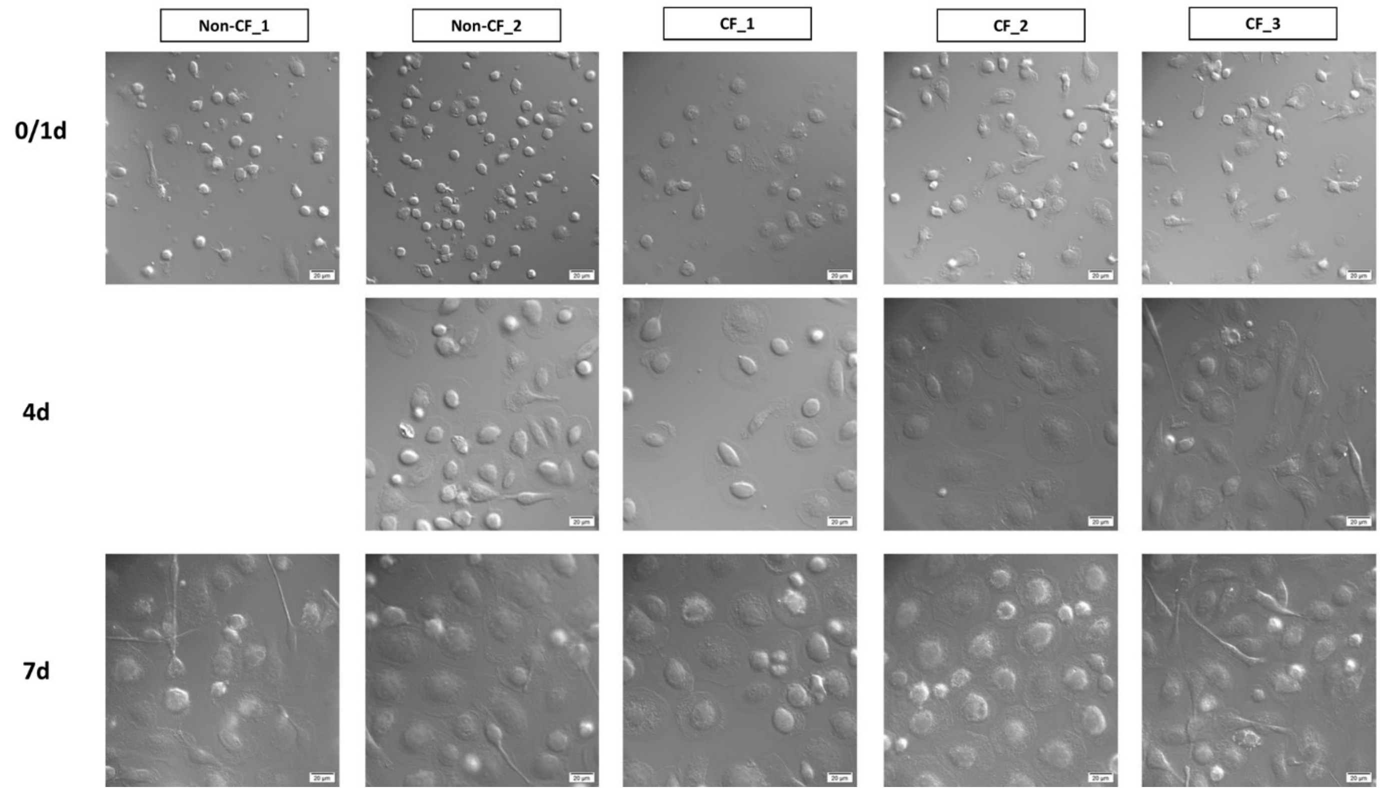

Supplementary Figure S1: *Related to Figure 1. Microscopy images of non-CF and CF monocyte differentiation.* CF (n = 3) and non-CF (n = 2) monocytes were imaged over the course of differentiation with 100 µg/ml M-CSF. Monocytes were imaged the day of seeding or the day after seeding (0/1d), on the fourth day (4d), and on the seventh day (7d). Scale bar is equal to 20 µm.

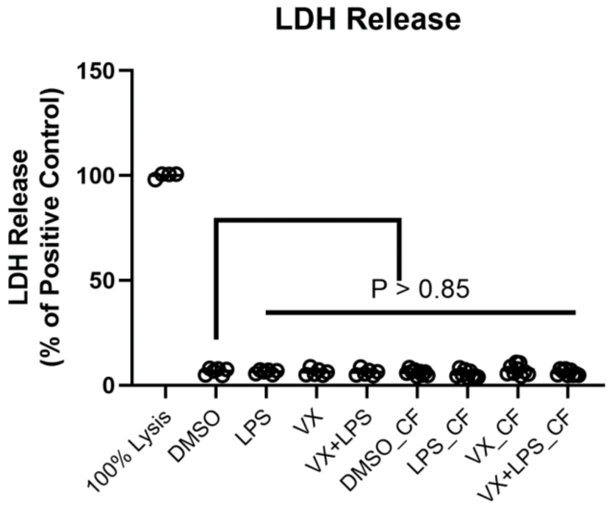

Supplementary Figure S2*: Related to Figure 1 and 2. LPS and CFTR modulators do not induce cytotoxicity in non-CF or CF MDMs.* LDH was measured in CF (n = 9) and non-CF (n = 6) supernatants after pretreatment for 48 hours with DMSO or modulators (ivacaftor [30 nM] and lumacaftor [3 µM], VX) followed by treatment for 24 hours with vehicle or LPS. Data analyzed using one-way ANOVA.

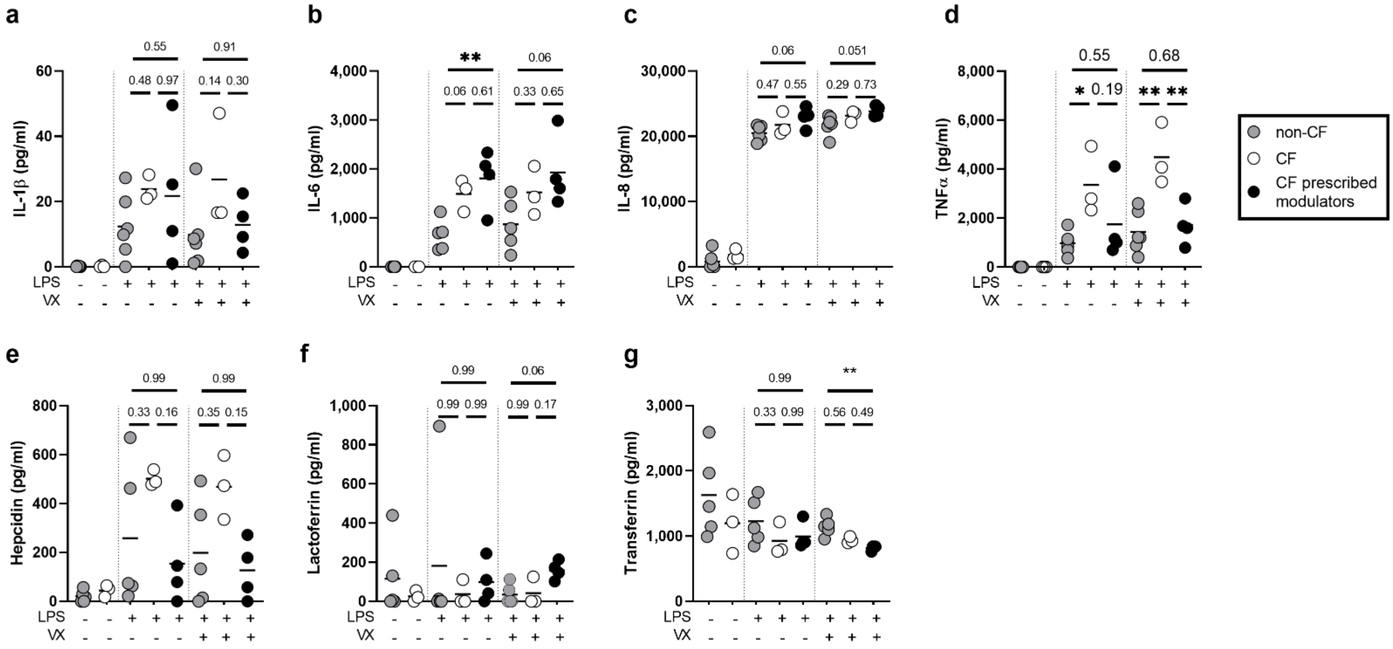

Supplementary Figure S3*: Related to Figure 1 and 2. Impact of prescribed use of CFTR modulators on cytokines and soluble iron-related protein secretion.* Data for CF subjects who were on modulators at the time of phlebotomy (n = 4) have been separated from data for CF subjects who were not (n = 3). Non-CF subject number for TNFα n = 5; IL-6, IL-8, and IL-1β n = 6; hepcidin, lactoferrin, and transferrin n = 5. After 48 hours pretreatment with vehicle or modulators (ivacaftor [30 nM] and lumacaftor [3 µM], VX), CF and non-CF MDMs were exposed to vehicle or LPS for 24 hours before supernatants were collected for ELISA for **(a)** IL-1β, **(b)** IL-6, **(c)** IL-8, **(d)** TNFα, **(e)** lactoferrin, **(f)** transferrin, and **(g)** hepcidin. (**a-d)** '*'

indicates P<0.05, '**' indicates P<0.01, one-way ANOVA **(a-c)** or Kruskal-Wallis test **(d). (e-g)** ('**' indicates P < 0.01, Kruskal-Wallis test).

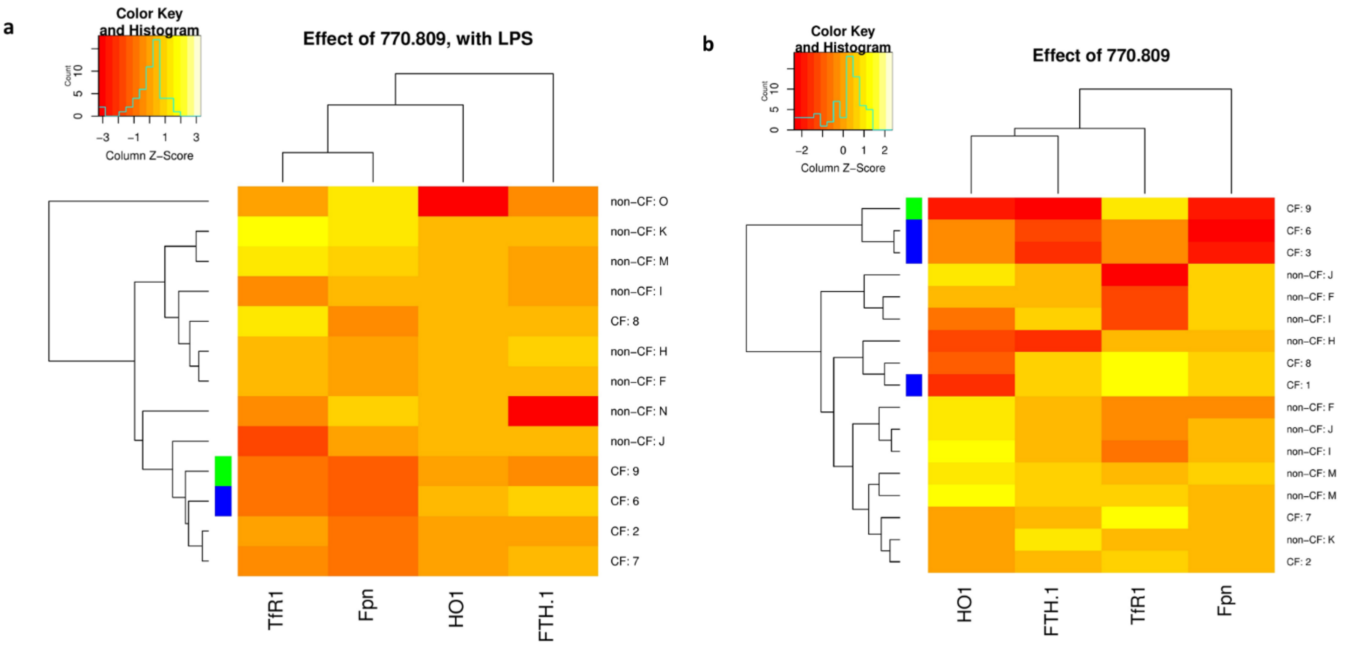

Supplementary Figure S4: *Related to Figure 2. The impact of prescribed modulator use on protein expression at baseline and in response to LPS.* MDMs were pretreated for 48 hours with vehicle (DMSO) or modulators (ivacaftor [30 nM] and lumacaftor [3 μM], VX), followed by 24 hours treatment with vehicle or LPS. Heatmaps were produced with the R package gplots[1] with the heatmap.2 function (gplots_3.0.1.2, https://CRAN.R-project.org/package=gplots). Columns indicate protein. Rows indicate subject, with arbitrary numbers and letters to identify CF and non-CF subjects. Columns are Z scaled and colors indicate the number of standard deviation (SD) units from the mean value for the whole column. For each column, red indicates values that are less than the mean of the protein measured and yellow indicates values that are higher than the mean of the protein measured. CF subjects who were taking clinically prescribed *in vivo* modulators at the time of phlebotomy are indicated on the left-hand side of the heat maps.

Subjects who were taking ivacaftor/lumacaftor are indicated in blue and subjects who were taking ivacaftor/tezacaftor are indicated in green. The deprograms indicate hierarchical clustering of similar proteins (columns) or samples (rows). **(a)** Related to Figure 2a, c, and d. The effect of modulator pretreatment on basal protein expression across subjects. **(b)** Related to Figure 2b, c, and e. The effect of both modulator pretreatment and LPS stimulation on protein expression across subjects.

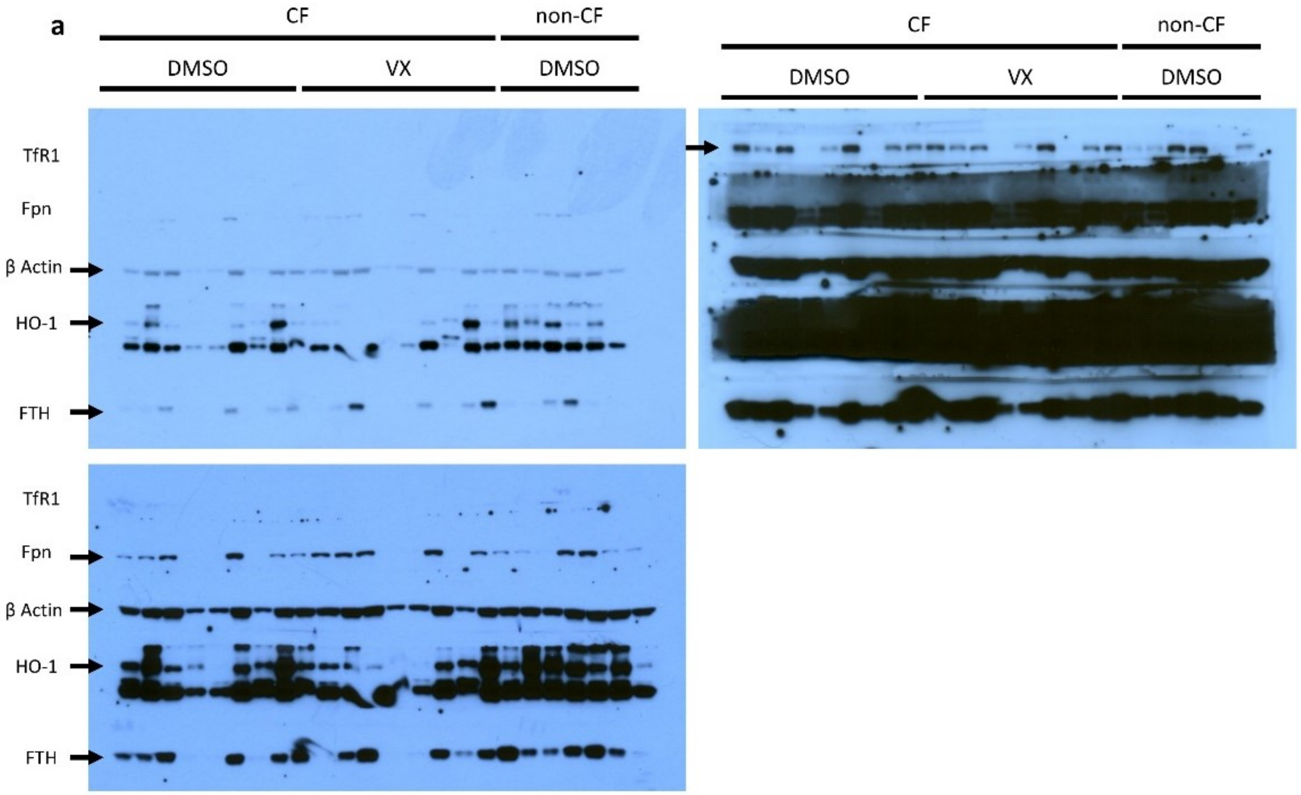
a
CF
non-CF
DMSO
VX
DMSO
TfR1
Fpn
β Actin
HO-1
FTH
CF
non-CF
DMSO
VX
DMSO

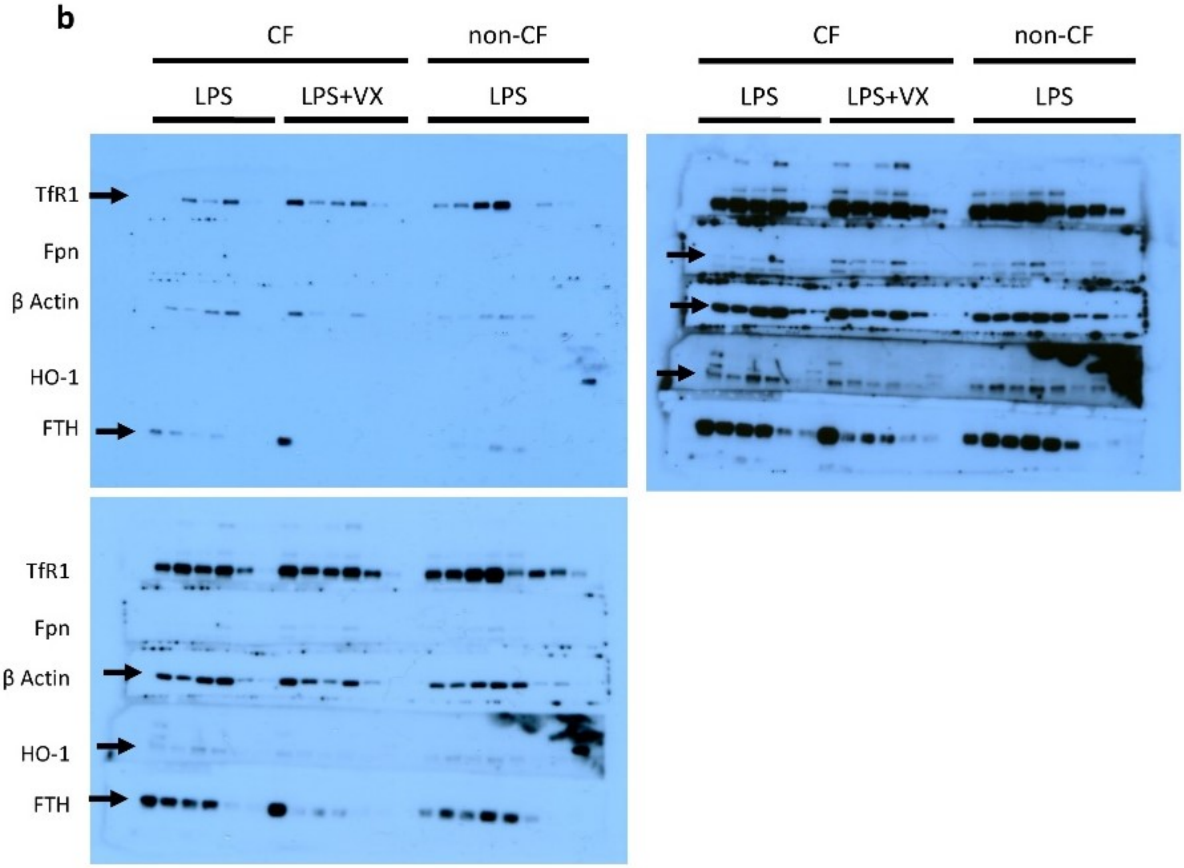
b
CF
non-CF
LPS
LPS+VX
LPS
TfR1
Fpn
β Actin
HO-1
FTH
CF
non-CF
LPS
LPS+VX
LPS

64

Supplementary Figure S5: *Related to Figure 2. Whole western blot images.* **(a)** Whole images of western blots prior to conversion to 8-bit format for densitometric analysis. Black arrows indicate use of cropped image in Figure 2a. **(b)** Whole images of western blots prior to conversion to 8-bit format for densitometric analysis. Black arrows indicate use of cropped image in Figure 2b.

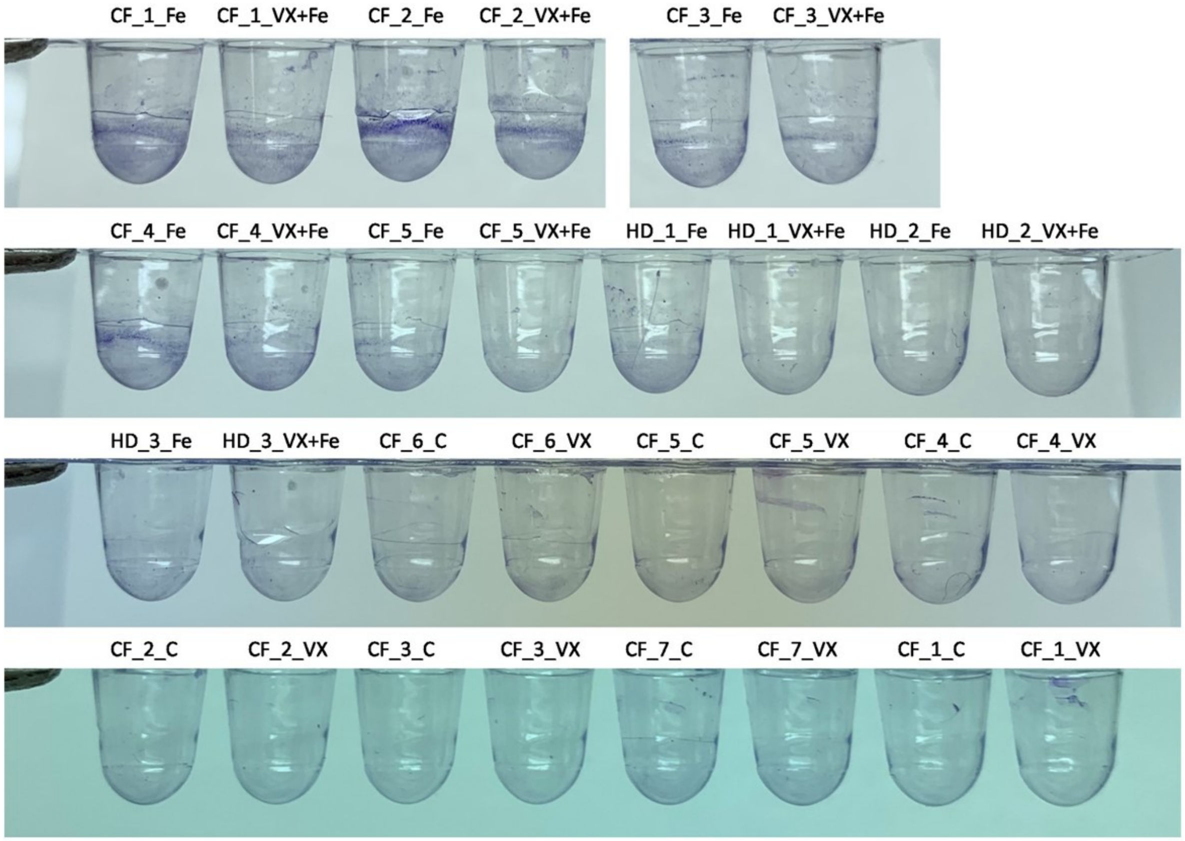

Supplementary Figure S6: *Related to Figure 4. All images for biofilm formation assay.* Representative images of biofilm formation from each subject and group tested. Images were taken at the same time. Arbitrary numeric labels do not correspond to those from Figure 2 or Supplementary Figure S4. CF ("CF", n=5) and non-CF ("HD", n=3) MDMs were pretreated for 48 hours with vehicle (DMSO) or modulators (ivacaftor [30 nM] and lumacaftor [3 µM], VX). Conditioned media was collected after an additional 48 hours with 30 µM $FeCl_3$ and used for bacterial biofilm formation assay.

1 Gregory & Warnes, R. (ed Ben Bolker) (https://CRAN.R-project.org/package=gplots, 2020).
